# Seventy-Seventh Annual Commencement

**Published:** 1923-06

**Authors:** 


### PROGRAM OF THE SEVENTY-SEVENTH ANNUAL COMMENCEMENT OF THE OHIO COLLEGE OF DENTAL SURGERY

**At the Odeon, Thursday, June 7, 1923**

MUSIC—March, "The Stars and Stripes Forever". . *Sousa*

INVOCATION

MUSIC—Overture, "The Bridal Rose" . . . . . . . . . *Lavalee*

MUSIC—Intermezzo, "Quartette from Rigoletto" . . . *Verdi*

INTRODUCTORY REMARKS

PROF. H. T. SMITH, *Dean*

ADDRESS

REV. JOHN F. HERGET, CINCINNATI, OHIO

AWARDING OF DEGREES

CHARLES I. KEELY, D. D. S.
*President of Board of Trustees*

MUSIC—"Dearest, You're the Nearest To My Heart". *Akst*

MUSIC—"Saw Mill River Road" . . . . . . . . . . . . . . . *Tierney*

MUSIC—Waltz, "Wonderful One" . . . . . . . . . . . *Whiteman*

ADDRESS FOR THE GRADUATING CLASS

EDWIN M. GODBY, D. D. S., LOGAN, W. VA.

VOCAL SOLOS—Selected Ballads { My Irish Rose / Down Among the Sleepy Hills of Tennessee }

RICHARD PAVEY

MUSIC—Selection, "Southern Airs" . . . . . . . . . . . . *Lampe*

AWARDING OF PRIZES

MUSIC—March, "Men of Valor" . . . . . . . . . . . . . . . . *Klohr*

*Music by Sibcy-Hofer Orchestra*

### GRADUATING CLASS, 1923, OF OHIO COLLEGE OF DENTAL SURGERY

Walter Benedict Albertson.....................East St. Louis, Ill.
J. Russell Anderson.............................Ernest, Pa.
James Rouse Askew..............................Butler, Ky.
Clyde Ray Baker..........................Fairmont, W. Va.
Harold S. Barnheiser........................Lewisburg, Ohio
James Foster Branch.............................Akron, Ohio
Glen B. Donoho..................................Fulton, Ky.
Kenneth M. Dunavent..........................Owenton, Ky.
Carroll J. Fairo.................................Butler, Ky.
Miriam Funk Warm.........................Cincinnati, Ohio
Edwin M. Godby...............................Logan, W. Va.
Arthur H. Goe..............................Weston, W. Va.
Emmet F. Gallagher..........................Cleveland, Ohio
Ormond T. Greenland........................Cincinnati, Ohio
Clarence J. Herman..........................Lewisburg, Ohio
Benjamin F. Hoagland.......................Cincinnati, Ohio
Mary F. Hoagland...........................Cincinnati, Ohio
William Cecil Hough.....................Fort Thomas, Ky.
Thomas Edward Hughes.......................Hamilton, Ohio.
Paul Thomas Jacoby........................Springfield, Ohio
Holly C. Jarvis........................Little Otter, W. Va.
Gordon Henry Johnson........................Hamilton, Ohio
Edward Horace Jones.......................Cincinnati, Ohio
Harold E. Kerr...............................Meadville, Pa.
Eloise Constance Kinney....................Cincinnati, Ohio
G. William Lavender......................Cabin Creek, W. Va.
Robert Thomas Linfert......................Cincinnati, Ohio
Fred T. Loukes.........................Port Henry, N. Y.
Roscoe A. McGinnis.........................Cincinnati, Ohio
Anna Marie McCormick..........................Xenia, Ohio
Charles Edwin Marshall..................Grantsville, W. Va.
C. Lee Martin............................Cedar Grove, W. Va.
Murdoch M. Miller..............................Canton, Ohio
J. Bernard Moses, Jr..........................Crestline, Ohio
J. Toy Nelson..............................Bluefield, W. Va.
Frank Stanley Patterson.....................Greensburg, Pa.
William W. Pollock..........................Hinton, W. Va.
R. Roscoe Powell...........................Greensburg, Ind.
Gordon Bernard Rader.....................Charleston, W. Va.
Karl J. Rhinehart...........................Lewisburg, Ohio
Arthur William Rossfeld.........................Lima, Ohio
Fred G. Ruble...................................Piqua, Ohio
Leon Saks..................................Cincinnati, Ohio
Wilbert John Schneider.......................Hamilton, Ohio
Troy M. Scurlock..............................Jackson, Ohio
Charles H. Shaw......................Sugartree Ridge, Ohio
Alvin A. Slutz.............................Cincinnati, Ohio
Jacob Soldovnick.................................Chicago, Ill.
Hoyt A. Starks..................................Osgood, Ind.
Frank O. Turner..............................Covington, Ky.
Dennis R. Vaughan......................Willow Bend, W. Va.
William F. Vosseler........................Cincinnati, Ohio
Elmer A. Welp...............................Covington, Ky.
Herbert S. Wiatt............................Gloucester, Va.
Floyd Elam Widger.............................Vandalia, Ill.
Ellsworth Leonard Wood.....................Cincinnati, Ohio

### PERSONNEL OF THE OHIO COLLEGE OF DENTAL SURGERY

### Board of Trustees

C. I. KEELY, D. D. S., *President*
DAVIS L. JAMES, *Vice-President*
ALBERT H. MORRILL, *Secretary*
H. T. SMITH, D. D. S., *Treasurer*
STANLEY G. BURT
REV. DUDLEY W. RHODES
ALBERT J. BELL, M. D.
ATLEY S. HENSHAW
CLINTON G. GALWAY

### Faculty and Instructors

HENRY T. SMITH, D. D. S., DEAN
*Professor of Dental Pathology and Principles of Operative Dentistry*

ATLEY S. HENSHAW, VICE-DEAN and REGISTRAR
*Professor of Physics, Drawing and English*

J. N. MYERS, A. B., D. D. S., SUPERINTENDENT OF CLINICS
*Professor of Prosthetic Technics and Clinical Prosthetics*

WILLIAM J. GRAF, B. A., M. D.
*Professor of Physiology and Physical Diagnosis*

CARL H. STRICKER, D. D. S., SECRETARY OF THE FACULTY
*Professor of Prosthetic Dentistry*

ARTHUR C. GEWERT, M. D., D. D. S.
*Professor of Anatomy*

ROBERT M. SCHELL, A. B., D. D. S.
*Professor of Oral Surgery, Anaesthetics and Exodontia*

GEORGE F. WOODBURY, D. D. S.
*Professor of Operative Dentistry, Crown and Bridge Work and Ethics*

LOUIS F. WERNER, PH. G., A. B., A. M., PH. D.
*Professor of Chemistry, Materia Medica and Pharmacology*

SARKIS KRIKORIAN, D. D. S.
*Assistant Professor of Crown and Bridge Work and Clinical Dentistry*

B. E. BALDRIDGE, D. D. S.
*Assistant Professor of Clinical Dentistry*

H. O. WALL, D. D. S.
*Assistant Professor of Clinical Dentistry*

B. A. SCHNEDL, D. D. S.
*Professor of Orthodontia*

J. HERMANIES, M. D.
*Instructor in Histology, Pathology and Biology*

SAMUEL RAFISH, D. D. S.
*Instructor in Dental Anatomy Technic and Operative Dentistry*

E. R. BADER, M. D., PH. G.
*Instructor in Radiology*

ALBERT H. MORRILL, A. B., LL. D.
*Lecturer on Dental Jurisprudence*

CHARLES I. KEELY, D. D. S.
*Visiting Supervisor of Clinical Dentistry*

---

L. E. CUSTER, B. S., D. D. S. . . *Lecturer, Dental Electro-Therapeutics*

ALBERT J. BELL, A. B., M. D.
*Lecturer, Pediatrics in Relation to Dentistry*

SIDNEY J. RAUH, D. D. S. . . . . . . *Instructor in Public-School Dentistry*

WYLIE MCLEAN AYRES, A. B., M. D.
*Instructor in Ophthalmology in Relation to Dentistry*

JOHN B. BOUTET, D. D. S. . . . . . . . . . . . . . . *Instructor in Periodontia*

